# Effectiveness of Autograft and Allograft Transplants in Treating Athletic Patients With Osteochondral Lesions of the Talus

**DOI:** 10.7759/cureus.29913

**Published:** 2022-10-04

**Authors:** Jake Vogel, Varun Soti

**Affiliations:** 1 Psychiatry, Lake Erie College of Osteopathic Medicine, Elmira, USA; 2 Pharmacology and Therapeutics, Lake Erie College of Osteopathic Medicine, Elmira, USA

**Keywords:** sports persons, active-duty military personnel, osteochondral allograft transplantation, autologous osteochondral transplantation, osteochondral lesions of the talus

## Abstract

Osteochondral lesions of the talus (OLTs) represent 50% of ankle sprains and are most common in athletes who play competitive sports or are on active military duty. OLTs can cause significant physical damage if left untreated and may inflict financial burdens and mental health issues. Over the years, replacement surgeries, mainly autologous osteochondral transplantation (AOT) and osteochondral allograft transplantation (OAT), have become instrumental in treating OLTs. However, these procedures’ effectiveness in returning to full fitness to resume competitive sports or active duty has not been well-established. This systematic review attempts to help this population cohort better understand OLTs and highlight the existing clinical evidence on AOT and OAT effectiveness in treating such patients. We performed a literature search between March 2022 through September 2022 following the Preferred Reporting Items for Systematic reviews and Meta-Analyses guidelines. Of eligible studies evaluating surgical outcomes of AOT and OAT in sportspeople and active-duty military personnel, 86% of patients who received AOT returned to competitive sports or active duty compared to 61% who received OAT. Additionally, on average, patients who underwent AOT returned to full fitness in five months rather than in 16 months for those who underwent OAT. As highlighted in this review, the limited evidence indicates that AOT may lead sportspeople and active-duty military personnel to return to pre-injury levels and resume athletic activities sooner. It is challenging to assume the same for OAT, given the limited studies in athletic cohorts with OLTs. Nevertheless, AOT and OAT are crucial surgical options that can significantly benefit competitive sportspeople and military personnel in resuming their careers.

## Introduction and background

Ankles are the most frequently injured parts of the human body. These injuries can significantly impact careers, especially for those who play professional sports or are in other professions such as the military, where high-intensity athleticism is integral. At least nine in 10,000 athletes suffer ankle injuries during competitive events [1]. Approximately 40% of ankle injuries and 50% of all ankle sprains occur during athletic activities [2]. If these injuries worsen, they can develop into osteochondral lesions of the talus (OLTs) [3]. Athletes who sustain OLTs can have shortened sporting careers and may not perform to the optimum level post-recovery. Hence, they may not be able to continue to play for the length of time they otherwise would, resulting in earlier retirements.

Through electronic communication, we reached out to some National League Football (NFL) players, including* Mr. Justin Quintin Reid*, a current NFL player starting strong safety for the Kansas City Chiefs, and *Mr. Christopher Michael Gronkowski*, who formally played for Dallas Cowboys, Indianapolis Colts, and Denver Broncos.

*Mr. Reid* candidly spoke about the difficulty “to pinpoint the exact source or the depth” of these injuries. He also revealed that “it is not uncommon to misdiagnose.” However, once there is a diagnosis, he stressed that it is vital for the player(s) to have faith in his physician “to make a plan, buy into it, and stick to it.” *Mr. Reid* identified the “mental aspect” of these physical injuries as “the most challenging” for him and other athletes. He described the internal pressure a player feels “when sidelined. It makes you feel like you are not part of the team.” He explained the internal pressure stems from the fact that high-performance athletes consider playing as part of their identity. It can only be maintained and fulfilled if they continue to play.

*Mr. Reid* stated that, in addition, there is external pressure, which comes from losing “millions if not billions of dollars.” The player’s injury adversely affects teams, coaches, managers, and fans. Due to these factors, a player, *Mr. Reid *said, feels the need to rush through the rehabilitation process and get back onto the field. “Often, athletes may be mentally confident they are ready to go when their bodies are not.”

*Mr. Gronkowski*, having suffered a torn pectoral muscle and high-grade ankle sprain, echoed the similar sentiment as *Mr. Reid* did on hastening the rehabilitation process. He frankly remarked, “as a bubble player,” he felt the “internal pressure to perform and work through injuries.” “Because anytime you are not on the field, you have a good chance of losing that job.” Although there was no push from his team and the NFL, *Mr. Gronkowski* felt the need to get back on the field as early as possible.

Hundreds of athletes and military personnel can identify with the experiences and perspectives of *Mr. Reid* and *Mr. Gronkowski*. It brings into light the conundrum athletes face when they suffer from OLTs. Due to the lack of knowledge about OLTs, they may struggle to make the right decision for their treatment plans, including surgical procedures and rehabilitation. It can limit them from giving themselves the best chance of success post-injury. Although it is difficult to fathom, several orthopedic practitioners may find it challenging to treat OLTs effectively. They may get caught up in choosing the best surgical option to get their patients back to optimum playing or working level.

Over the years, two specific replacement surgical procedures have become instrumental in treating OLTs. These include autologous osteochondral transplantation (AOT) and osteochondral allograft transplantation (OAT) [4]. Therefore, it is vital to understand the clinical advantage of these surgical options to achieve the best patient outcomes. Thus, this review showcases the clinical benefits of AOT and OAT in treating OLTs.

This review aims to advance the understanding of OLTs’ in players and military personnel who are more prone to developing OLTs. Furthermore, it presents clinical evidence to highlight the effectiveness of AOT and OAT in treating athletic cohorts with OLTs.

## Review

Methods

Data Sources and Study Selection

**Figure 1:**
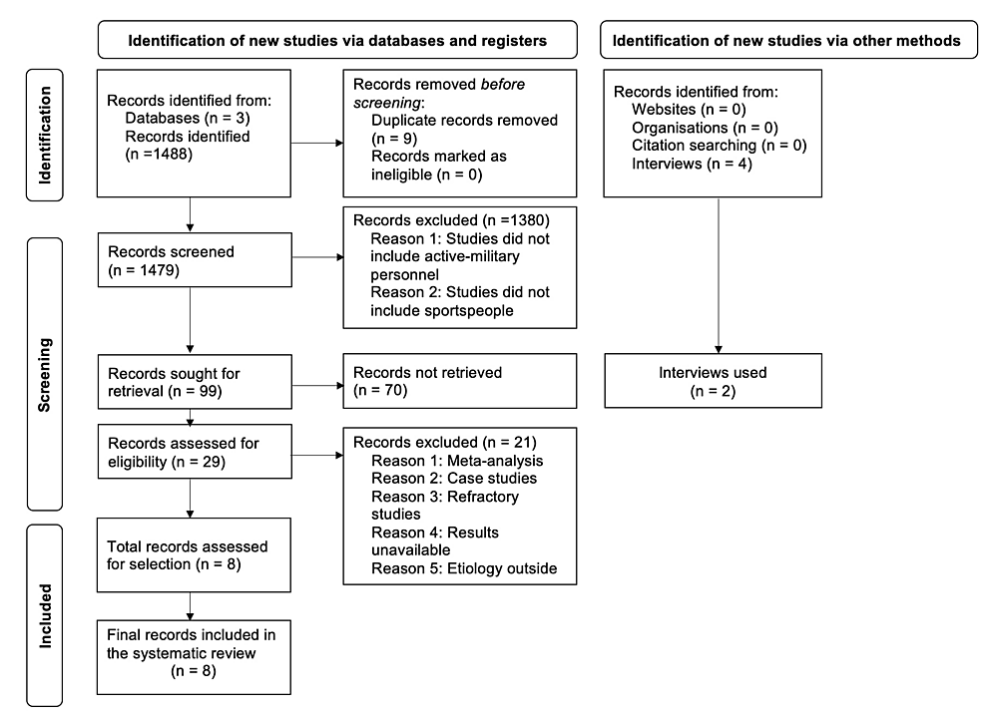
Literature search and selection process. This systematic review by following the Preferred Reporting Items for Systematic Reviews and Meta-Analyses guidelines [5] utilized PubMed, BioMed Central, and Clinicaltrials.gov to search for clinical studies on autologous osteochondral transplantation and osteochondral allograft transplantation in treating the athletic population with osteochondral lesions of the talus. The keywords were limited to “Autograft versus Allograft Ankle,” “Osteochondral Lesions of the Talus,” “Osteochondral Lesions of the Talus Autograft,” and “Osteochondral Lesions of the Talus Allograft.” By using filters and inclusion criteria, including articles written in English, and complete clinical studies with select patient demographics (sportspeople and active-duty military personnel), we selected relevant studies.

We conducted a literature search between March through September 2022 on PubMed, BioMed Central, and Clinicaltrials.gov databases per the Preferred Reporting Items for Systematic Reviews and Meta-Analyses guidelines [5] (Figure 1). We selected relevant studies written in English and assigned a level of clinical evidence per the previous literature [6].

Data Extraction

On PubMed, the keywords “Autograft versus Allograft Ankle” and “Osteochondral Lesions of the Talus” in led to the retrieval of 19 and 28 studies, respectively. “Osteochondral Lesions of the Talus Autograft” resulted in seven studies. “Osteochondral Lesions of the Talus Allograft” resulted in two studies. However, on BioMed Central, “Osteochondral Lesions of the Talus Autograft” yielded 14 records. “Osteochondral Lesions of the Talus Allograft” also produced 14 articles. On Clinicaltrials.gov, “Osteochondral Lesions of the Talus” showed 15 studies.

Osteochondral lesions of the talus

The talus comprises three specific regions that make up the bone: the body, neck, and head. Osteochondral lesions typically occur within the dome of the body of the talus [3]. The talus has a unique aspect to its anatomy that, when an injury occurs, it limits the healing potential for the area. This is because articular cartilage covers 60% of its surface, limiting its vascular supply. This creates watershed areas throughout the talus and limits its healing potential. OLTs refer to any defect involving the talus’ articular surface and subchondral bone. They make up to 73% of acute ankle injuries and 50% of ankle sprains [3-4]. There is an association between OLTs and a shearing force that occurs with severe dorsiflexion and ankle inversion [3]. The high frequencies of OLTs associated with athletic activities result from the stress that occurs during sprinting, sudden direction changes, tackling, and kicking [7].

Diagnostic Procedures and Classification of OLTs

The first step in diagnosing OLTs is imaging the ankle. The initial modality for suspected OLTs is a plain radiograph with standard ankle views. There is better visualization of talar dome lesions when the foot is pronated 15 degrees, and the X-ray beam is angled 75 degrees cephalad. To further detect defects in the cartilage or displaced lesions, bone scintigraphy is a better option. It has 96% specificity and 94% sensitivity to screen abnormalities such as occult osteochondral lesions [8]. Other imagining modalities are computed tomography and magnetic resonance imaging (MRI) [9].

After imaging the ankle, the next step is classifying the lesion. Hepple et al. [10] classified OLTs into the following categories. Stage I represents articular cartilage damage. Stage IIa shows articular cartilage damage with underlying fracture and bony edema. Stage IIb is similar to stage IIa but without bony edema. Stage III represents a detached but displaced osteochondral fragment. Stage IV shows a displaced osteochondral lesion, and stage V subchondral cyst formation [9].

Surgical management of OLTs

Surgical treatments involve reparative and replacement procedures. Reparative procedures are bone marrow stimulation, bone marrow-derived cell transplantation, and autologous matrix-induced chondrogenesis. Replacement procedures include AOT and OAT [1].

Autologous Osteochondral Transplantation

AOT is a cartilage replacement procedure to reproduce the mechanical and structural properties of the hyaline articular cartilage native to the talus. AOT is performed by harvesting an osteochondral graft from the non-weight-bearing portion of the ipsilateral knee and inserting a cylindrical osteochondral graft into the prepared lesion site on the talus. The procedure begins with performing a tibial osteotomy to visualize the lesion and removing the talar lesion trephine with controlled mallet taps. Next is bone marrow stimulation to the surrounding healthy bone, followed by an overdrill to make the recipient sit slightly longer than the harvested graft. The next step is harvesting the osteochondral graft from the non-weight-bearing portion of the ipsilateral femoral condyle. Finally, the procedure completes with the insertion of the osteochondral graft into the created recipient site [1].

Osteochondral Allograft Transplantation

OAT involves grafting a cadaver’s articular cartilage and bone into a recipient. The procedural steps include excising the talus’ diseased portion using reciprocating and microsagittal saws and cold saline irrigation solution. Next, measure the dimensions of the talar lesion at least twice to obtain accurate measurements and clamp the allograft talus carefully with bone-holding forceps. Find the diseased talus lesion’s location on the allograft and mark the dimensions from the diseased talus. Afterward, extract the graft from the allograft, wash it with a sterile saline solution, and place it into the recipient site. Match the graft’s articular surface with the articular surface of the native talus and secure the graft with one or two 1.5 to 2.0-millimeter diameter solid screws [11].

AOT and OAT in treating cohorts of athletes and active-duty military personnel

Nguyen et al. [12] were among the renowned orthopedic researchers who studied AOT (Table 1). They investigated AOT as a treatment modality in treating osteochondral lesions greater than 150 square millimeters (mm^2^) in professional and amateur athletes and if they could successfully return to sports post-AOT. They enrolled 38 athletes who had OLTs (lesions greater than 150 mm^2^) and a preinjury score of 6 or greater on the Tegner Activity Level Scale (TALS) [12].

**Table 1:** Key clinical studies evaluating AOT effectiveness in treating OLTs in the Athletic population. The clinical studies meeting the inclusion criteria of this review showed the benefits of AOT in the treatment of OLTs in cohorts of athletic populations, including professional and amateur athletes. These studies utilized AOFAS, FAOS, Hannover ankle scores, SF-12, and VAS questionnaires to assess the improvements in patients following surgery. AOFAS, American Orthopaedic Foot and Ankle Society ankle-hindfoot score; AOT, autologous osteochondral transplantation; FAOS, Foot and Ankle Outcome Scores; mm, millimeters; OLT, osteochondral lesions of the talus; p, probability variable; SF-12, Short Form-12; VAS, visual analog scale

Authors	Level of evidence	Sample size	Mean age of patient at the time of surgery (years)	Follow-up time (months)	Mean size of lesion (mm^2^)	Mean time to return to sports post-AOT (months)	Assessment tool	Findings
Nguyen et al. 2019 [12]	II	38 patients	26	44.71	249	8 (amateur athletes); 6 (professional athletes)	VAS and FAOS	VAS scores decreased from 4.53 to 0.63 post-AOT (p = 0.002). All five aspects of FAOS (sports, pain, symptom, ADL, and QoL) improved post-AOT (p < 0.001).
Fraser et al. 2016 [13]	II	36 patients	31	71	133	6	AOFAS	AOFAS scores improved from 65.5 to 89.4 post-AOT (p = 0.01). About 67% of patients scored between 90 and 100 (excellent). About 86% of patients returned to pre-injury levels within 12 months post-AOT.
Paul et al. 2012 [14]	II	131 patients	31	60	Not reported	Not reported	Number of sport and recreational activities pre- and post-AOT. Duration of sports and recreational activities before and after surgery (hours). VAS	There was no change (p = 0.053) in patients’ participation in the number of sports post-AOT. There was no change (p = 0.052) in patients’ duration of sports activities post-AOT. VAS scores decreased from 6.3 to 2.7 (p < 0.001).
Saxena and Eakin 2007 [15]	II	44 patients	36	32	Not reported	5	AOFAS	AOFAS scores improved from 46.1 pre-AOT to 93.4 post-AOT (p < 0.001).
Kennedy and Murawski 2011 [16]	II	72 patients	34	28	11.2	3	FAOS and SF-12	FAOS scores improved from 52.67 pre-AOT to 86.19 post-AOT. No statistical analysis was available. SF-12 scores improved from 59.40 pre-AOT to 88.63 post-AOT. No statistical analysis was available.
Gautier et al. 2002 [17]	II	11 patients	30	24	18 x 10	Not reported	AOFAS and Hannover ankle scores	The average AOFAS score post-AOT was 92.09 (p < 0.001). The average Hannover ankle score was 91.55. 81.8% reported excellent outcomes and 18.2% reported good outcomes (p < 0.001).

Of the 38, 11 were professional athletes, and 27 were amateurs. They were male with a mean age of 26 years. They were assessed for their ability to return to sports at follow-up at a minimum of two years post-AOT. Their return to sports was considered *full* if they could compete at pre-injury levels, *partial return* if their functional competence were lower, and a lower TALS score than before the injury, and *no return* if they did not return or could not compete. For professional athletes, *full return* included the time they took after the surgery to participate in training camps and return to playing at the optimum level within their team squads. Of the 38 patients, 33 (86%) returned to their respective sports and competed at levels they did before the injury. All 11 professional athletes made a *full return*; four amateur athletes had a *partial return*, and one had *no return* [12].

In addition, Nguyen et al. [12] utilized the visual analog scale (VAS) to assess pain and a Foot and Ankle Outcome Score (FAOS) pre-AOT and 12 months post-AOT. There were significant improvements in VAS scores, as they reduced from 4.53 pre-AOT to 0.63 post-AOT (p = 0.002). Moreover, FAOS scores remarkably improved post-AOT: sports and recreation had a mean score improvement of greater than 49.2 (p < 0.001). The pain scores substantially improved with a mean score of greater than 36.5 (p < 0.001). Scores for symptoms, activities of daily living, and quality of life increased prominently, with mean scores of greater than 19.3 (p < 0.001), 32.5 (p < 0.001), and 43.5 (p < 0.001), respectively. Nguyen et al. [12] demonstrated that AOT is an effective surgical option for treating OLTs in professional athletes. However, these observations could have been widely generalized if their study had a control group.

To further understand AOT's effectiveness in treating OLTs, Fraser et al. [13], in a retrospective analysis, examined the return to sports outcomes for 36 athletes who had previously undergone AOT. Among the 36 patients, 21 played sports professionally, and 15 were amateurs. Their average age was 31 years, and the ratio of males to females was 2:1. Before AOT, all patients underwent a physical examination. Also, all study participants had a pre-operative MRI scan [13].

Fraser et al. [13] utilized the American Orthopedic Foot and Ankle (hindfoot) Score (AOFAS) to assess surgical clinical outcomes. A grade between 90 and 100 indicated an *excellent* outcome. A *fair* outcome was tantamount to a score between 70 and 79, and a score below 70 predicted a *poor* outcome. The researchers determined the return to sport as *full return*, *restricted return*, and *no return* by following up with patients at 12 and 24 months post-AOT. They defined *full return* to sport as a function of performance at pre-injury levels, with any possible symptoms experienced not interfering with their performance.* Restricted return* was returning to a lower level of athletic performance than pre-injury, and *no return* included athletes unable or unwilling to return to sport [13].

Following AOT, overall AOFAS scores improved from the mean pre-operative score of 65.5 to 89.4 (p = 0.01), with 67% of AOFAS scores falling under the *excellent* category, 19% were considered *good*, 11% *fair*, and 3% *poor*. There were no significant differences in patient outcomes based on gender, age, lesion size, and the number of osteochondral grafts. The mean time to return to full activity post-AOT was six months, with 86% of patients returning to pre-injury levels by 12 months [13].

Professional athletes had a 10% higher rate of *full return* than amateurs. Of the 36 patients, four (11%) encountered donor site morbidity from the harvested knee. Three of these four patients described discomfort with squatting and sport-specific movements. However, no patients experienced any functional limitations in the donor's knee. It was worth noting that although 86% of the patients returned to pre-injury levels post-AOT, the sample size was relatively small (N = 36). Another possible limitation of the study was using only a single tool to assess patient outcomes before and after surgery [13]. Nevertheless, these remarkable results showed the potential of AOT in treating OLTs in professional sportspeople.

In yet another retrospective evaluation of AOT in treating sportspeople with AOT and their return to high-intensity sports, Paul et al. [14] evaluated records of 158 OLT patients who underwent AOT. Of the 158, 131 (82.9%) had complete records. Of these 131, 81 were male, and 30 were female. Their mean average age was 31 years. Moreover, all had completed a previously applied sports and activity questionnaire to assess participation in 20 sports and recreational activities during specific times before and after AOT [14].

During the year before undergoing AOT, 83.8% of patients participated in an average of 3.7 different sports. The weekly frequency of sports activities did not change significantly post-AOT compared to pre-AOT, with 2.2 sports activities pre-AOT compared to 1.7 post-AOT (p = 0.053). Furthermore, the sports activity duration also did not considerably change, with sports activities decreasing from 5.1 hours pre-AOT to 4.2 hours post-AOT (p = 0.052) [14].

Furthermore, VAS was used to evaluate pain, and the Lysholm knee scale was employed to assess the knee harvested from the donor osteochondral graft. The VAS scores reduced significantly from 6.3 (pre-AOT) to 2.7 (post-AOT), p < 0.001. Also, the mean Lysholm score of this study group was 88 points. Around 43% (56) patients reported *excellent* results, with a score between 98 and 100 points, and 13% (17) reported *good* to *excellent* results, with a score between 92 and 97 points. Moreover, 20% (26) had *fair* to *good* results (scores between 82 to 91 points), and 15% (20) had *fair* results, with 10 patients scoring above 75 points and another 10 scoring below 75. Only 9% of study patients had poor results. Interestingly, there were no such changes in the number or duration of sports activities post-AOT compared to pre-AOT. However, VAS scores showed statistically significant improvements (p < 0.001) post-AOT [14].

The lack of substantial changes in sports activities could be due to many factors, for example, the patients’ age, AOT’s effectiveness, surgeons’ competency and experience, and the rehabilitation duration, which the researchers did not consider. A crucial limitation was also the retrospective nature of this study. It required patients to recall clinical and sports activity-related information dating back several years [14] that might not be accurate.

Saxena and Eakin [15] were interested in assessing athletes’ ability to return to athletic activities following injuries to the talus. They evaluated data of 44 athletes with talar injuries. The study participants had undergone either microfracture surgery or AOT from 1997 through 2003. Among the 44 patients, 20 received AOT as their surgical treatment, while the remaining 24 received microfracture surgical repair. Of the patients who received AOT, the average age was 36 years; 18 had high levels of athletic activity, including playing collegiate sports and marathon running. The other 26 engaged in multiple sports recreationally. Before receiving AOT, the researchers [15] evaluated patients using the AOFAS hindfoot rating score. Patients who underwent AOT had a baseline score of 46.1. At the re-evaluation post-AOT, the AOFAS scores, on average, ranged between 47.3 and 93.4 points (p < 0.001). The average return to activity post-AOT was 19.6 weeks.

While there was a significant increase in AOFAS scores post-AOT than pre-AOT, a major limitation, as noted by Saxena and Eakin [15], was a lack of specific documentation for the outcomes of those patients with high-demand activities following return to athletic activity.

In yet another study, Kennedy and Murawski [16] demonstrated the functional outcomes of AOT in 72 patients who were athletes, suffered from OLTs, and underwent AOT between 2005 and 2009. Of the 72 patients, 42 had been competing at some level of athletic sports before undergoing AOT. The mean age of study participants was 34 years, with 47 male and 25 female patients.

Kennedy and Murawski [16] assessed the effectiveness of AOT by utilizing FAOS and the Short Form-12 (SF-12) General Health Questionnaire before AOT and post-AOT. The average follow-up time for reassessment post-AOT of the patients was 28 months. At follow-up, the average FAOS score improved from 52.67 pre-AOT to 86.19 post-AOT. In addition, SF-12 scores improved from 59.4 pre-AOT to 88.63 post-AOT. Moreover, the average return to sports activity time was 13 weeks, with 40 athletes returning to previous activity post-AOT.

Despite remarkable improvements in assessment scores post-AOT and return to athletic activity in an impressive 13 week-period, Kennedy and Murawski [16] did not perform statistical analysis, which would have definitively established the significance of AOT in surgically treating athletic cohorts of patients.

To further describe AOT outcomes in athletic cohorts, Gautier et al. [17] retrospectively analyzed data from 11 patients who received AOT between 1996 and 1999. Of the 11 patients, eight were male and three were female. Six were involved in athletic sports, three played team sports, including soccer, two were competitive-level athletes, and one was a high-performance athlete. The other five participants participated in recreational sports and active walks. The average age of all study participants was 34 years and 25.9 years for males and females, respectively, and the average follow-up time post-AOT was 24 months [17].

Gautier et al. [17] utilized AOFAS and the Hannover ankle scores and compared post-AOT scores with baseline scores. The Hannover ankle scores range from 0 to 100. Excellent scores range from 85 to 104, good scores range from 65 to 84, satisfactory scores range from 35 to 64, and poor scores are below. At follow-up post-AOT, the average AOFAS score was 92.09, and the average Hannover ankle score was 91.55. Of 11 patients, nine reported excellent Hannover ankle scores the post-AOT, and two reported good scores compared to baseline scores (p < 0.001). Moreover, eight out of 11 patients scored more than 90 on AOFAS, with five scoring 100, one scoring 88, and two scoring 75 and 72, which were remarkedly higher than their baseline scores (p < 0.001). In addition, all patients returned to their previous level of sporting activities [17].

The clinical utility of AOT in successfully treating OLTs in athletic cohorts, yet again demonstrated by Gautier et al. [17], substantiates the need for future research endeavors featuring larger sample sizes.

On the other hand, Jackson et al. [7] were one of the two research groups (Table 2) that studied patient outcomes in athletic patients who underwent OAT. In one such study, Jackson et al. [7] specifically focused on assessing return to active duty and duty limitations caused due to operated ankle post-OAT. They enrolled soldiers with OLTs greater than 1.5 cm^2^, cystic lesions, and uncontained lesions on the shoulder of the talus or extending through its side wall [7].

**Table 2:** Available two clinical studies evaluating OAT’s effectiveness in treating OLT in the Athletic population. The only two clinical studies available showed the OAT’s benefits in treating OLT in members of the athletic population, such as professional athletes, amateur athletes, and active-duty military members. These studies utilized AOFAS and VAS questionnaires to assess the improvements in patients’ post-OAT. AOFAS, American Orthopaedic Foot and Ankle Society ankle hindfoot score; cm, centimeters; AOT, autologous osteochondral transplantation; mm, millimeters; OLT, osteochondral lesions of the talus; p, probability variable; VAS, visual analog scale

Authors	Level of evidence	Sample size	Mean age of patient (years)	Follow-up time (months)	Mean lesion size (cm^2 ^and mm^3^)	Mean time to return to sports post-OAT (months)	Assessment tools	Findings
Jackson et al. 2019 [7]	II	31 patients	33.77	21	1.37 cm^2^	21	Based on ability to return to duty	About 61% of patients were either in excellent or good categories post-OAT. Patients’ age (p = 0.167), size of lesion (p = 0.347), and cystic changes (p = 0.931) did not impact return to active-duty post-OAT.
Orr et al. 2017 [18]	II	8 patients	34.4	28.5	2247 mm^3^ (volume)	11	AOFAS; VAS	AOFAS scores improved from 49.6 to 73 post-OAT (p < 0.05). VAS scores decreased from 6.9 to 4.5 (p > 0.05).

The study reported results as *excellent*, *good*, or *poor*. Results were *excellent* if soldiers returned to active duty without restrictions. They were *good* if they returned to active duty with some restrictions and *poor* if they could not return to active duty. Physical therapy was initiated two weeks following the OAT and had patient follow-ups at six weeks, three and six months, and one and two years postoperatively. Of the 31 soldiers, 19 (61%) reported either *excellent* or *good* results, with 11 of the 19 reporting *excellent* results. However, 12 (39%) patients showed *poor* results post-OAT. When comparing patients who had *excellent* or *good* results with those who had *poor* results, there were no significant differences between the groups in terms of age (p = 0.163), lesion area (p = 0.347), or cystic changes (p = 0.931) [7].

Although Jackson et al. [7] demonstrated that 61% of the patient cohort with OLTs showed significant improvement and returned to active duty with or without restrictions after undergoing OAT [7], it is remarkably lesser than those who underwent AOT, as shown by Nguyen et al. [12], wherein about 86% athletes returned to high-intensity sports post-AOT [12], even though both studies were comparable in sample size with 31 [7] and 38 [12] patients, respectively.

In a retrospective analysis, Orr et al. [18] measured functional and occupational outcomes of active-duty military personnel with OLTs who underwent OAT. They looked at the records of eight patients with OLTs greater than 150 mm^2^ in size. Their average age was 34.4 years. Before undergoing OAT, all patients were assessed for pain and injury extent through VAS and AOFAS, respectively, to establish baseline scores [18].

Post-OAT, patients were followed up at an average of 29.5 months. Their average AOFAS score significantly improved from 49.6 (pre-OAT) to 72.8 (p < 0.05), with a considerable increase of 32%. The average VAS score substantially reduced from 6.9 (pre-OAT) to 4.5 post-OAT, with a substantial decrease of 35%. Of the eight patients, six returned to active duty, while two did not return [18]. 

Although the study by Orr et al. [18] showed remarkable improvement in patient outcomes measured in terms of VAS and AOFAS post-OAT, the sample size was minimal. It is in contrast with a retrospective analysis by Fraser et al. [13], showing about 97% had significant improvement in AOFAS scores ranging from fair to excellent, with 67% reporting excellent scores and returning to competitive sports and performing at pre-injury high-intensity levels post-AOT [13]. Moreover, post-OAT, the average time to resume active duty was 11 months [18] compared to six months when the majority of the athletes returned to their optimum sporting fitness post-AOT, as reported by Fraser et al. [13].

OLTs present a significant threat to athletes’ health and career, whether in competitive sports or professions where high-intensity training or athleticism is a prerequisite. As *Mr. Reid*, *Mr. Gronkowski*, and other players (not included in this paper) stressed that OLTs inflict physical damage, which can lead to fiscal and mental health problems. Therefore, they must clearly understand the injury and their surgical options.

It is crucial for such an exclusive group vulnerable to sustaining ankle injuries and developing OLTs to understand these injuries and different replacement surgical procedures thoroughly. It will make it far easier for them to make better healthcare decisions leading to better patient outcomes. In addition, it will further increase their chances of returning to optimum fitness and allow them to compete professionally or return to their active duty. It is equally vital for orthopedic surgeons and other professionals involved in the healthcare of these vulnerable groups of populations to keep abreast with the advantages and disadvantages of such procedures and tailor the treatment options to cater to the unique needs of their patients with OLTs.

**Figure 2:**
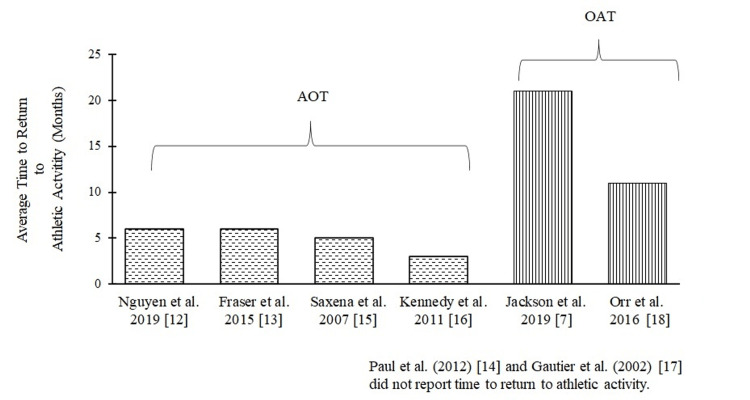
Time of return to active duty or competitive sports in the athletic population with OLTs post-AOT and OAT. This figure illustrates the time to return to active duty or competitive sports in studies conducted by Nguyen et al. [12], Fraser et al. [13], Saxena and Eakin [15], Kennedy and Murawski [16], Jackson et al. [7], and Orr et al. [18]. The average time to return to active duty or play competitive sports in athletic cohorts with OLTs undergoing OAT was 16 months. Athletic cohorts with OLTs undergoing AOT returned on average within five months. The other two studies by Paul et al. [14] and Gautier et al. [17] reviewed in this paper did not report the time to return to athletic activity following AOT and OAT, respectively. AOT, autologous osteochondral transplantation; OAT, osteochondral allograft transplantation; OLT, osteochondral lesions of the talus

A systematic review of the existing literature indicates that patients with OLTs undergoing AOT stand a better chance of a speedy recovery, gain optimum fitness similar to the pre-injury level, and return to competitive sports or active duty. Reviewing the existing studies that used AOT to treat athletic cohorts with OLTs, we found the rate of return to pre-injury levels was remarkable (86%) [9]. Moreover, after receiving AOT, the patient returned to full fitness for an average of five months [12-13,15-16]. This recovery duration is crucial for competitive sportspeople and military personnel (Figure 2).

Interestingly, only two studies [7,18] evaluated OAT’s effectiveness in treating athletic cohorts suffering from OLTs. Due to the lack of a number of studies on OAT in the athletic population, it is challenging to compare the duration of recovery and return to athletic activity post-OAT with post-AOT. Moreover, there was no clinical study that directly compared OAT with AOT. Also, the sample size of these studies was relatively smaller. Therefore, to establish the clinical superiority of AOT over OAT in treating the athletic population with OLTs, more clinical studies with a greater sample size directly comparing AOT with OAT are required.

Researchers must explore the longevity of an athlete’s or military personnel’s career following either type of replacement surgery for treating OLTs. It will be critical in helping such patients and their healthcare providers make better surgical decisions.

## Conclusions

As OLTs, a common ankle injury, continue to adversely impact the active population and, more specifically, people who play competitive sports or are in the military, patients constantly face dilemmas about making the right treatment choice to return to the field or active duty quickly. Also, it often brings financial burdens and mental issues. Therefore, their healthcare providers must help them pick the best treatment options that usually involve replacement surgical procedures. Over the years, AOTs and OATs have been the cornerstone of treating OLTs. For this purpose, we spoke with former NFL players. In addition, we systematically reviewed the current literature on AOTs and OATs, their success rates, and recovery duration to full fitness to return to active sports or duty. The existing clinical evidence indicates that AOT is effective in surgically repairing OLTs in athletic cohorts. Moreover, it helps them reach the optimum athletic fitness to pre-injury levels faster and has a quicker recovery time. OAT, like AOT, can also effectively treat such patients. However, it is challenging to compare recovery time and return to athletic activity post-OAT with post-AOT due to a limited number of studies on OAT. Returning to sports or active duty at the earliest is the priority for sportspeople and active-duty military personnel. Moreover, continuing to play competitively or perform duties after having undergone these surgical procedures at pre-injury levels for the desired length is critical. Therefore, studies further investigating the impact of these orthopedic procedures on the longevity of careers will provide a better understanding of the benefits of AOTs and OATs and better patient outcomes in the athletic population.
